# The *da1* mutation in wheat increases grain size under ambient and elevated CO_2_ but not grain yield due to trade‐off between grain size and grain number

**DOI:** 10.1002/pei3.10041

**Published:** 2021-03-13

**Authors:** Isabel Mora‐Ramirez, Heiko Weichert, Nicolaus von Wirén, Claus Frohberg, Stefanie de Bodt, Ralf‐Christian Schmidt, Hans Weber

**Affiliations:** ^1^ Leibniz Institut für Pflanzengenetik und Kulturpflanzenforschung (IPK) Gatersleben Germany; ^2^ BASF Innovation Center Gent Gent Belgium

**Keywords:** CO_2_ fertilization, *DA1*‐gene, grain number, grain quality, grain size, sink strength, trade‐off, wheat, wheat yield components, yield potential

## Abstract

Grain size is potentially yield determining in wheat, controlled by the ubiquitin pathway and negatively regulated by ubiquitin receptor DA1. We analyzed whether increased thousand grain weight in wheat *da1* mutant is translated into higher grain yield and whether additional carbon provided by elevated (e)CO_2_ can be better used by the *da1*, displaying higher grain sink strength and size. Yield‐related, biomass, grain quality traits, and grain dimensions were analyzed by two‐factorial mixed‐model analysis, regarding genotype and eCO_2_. *da1* increased grain size but reduced spikes and grains per plant, grains per spike, and spikelets per spike, independent of eCO_2_ treatment, leaving total grain yield unchanged. eCO_2_ increased yield and grain number additively and independently of *da1* but did not overcome the trade‐off between grain size and number observed for *da1*. eCO_2_ but not *da1* impaired grain quality, strongly decreasing concentrations of several macroelement and microelement. In conclusion, intrinsic stimulation of grain sink strength and grain size, achieved by *da1*, is not benefitting total yield unless trade‐offs between grain size and numbers can be overcome. The results reveal interactions of yield components in *da1*‐wheat under ambient and eCO_2_, thereby uncovering limitations enhancing wheat yield potential.

## 1 INTRODUCTION

Wheat (*Triticum* spp.) accounts for 30% of the global grain production and of 45% of the cereal nutrition, thus representing a major food crop species (FAOSTAT, http://www.fao.org/3/ca6030en/ca6030en.pdf). Despite significant breeding progress, yield levels of wheat remain below the required amounts to cope with the growing human population and with climate change consequences (Ray et al., 2013). Grain yield is the result of different components that interact in a complex and multiplicative manner (Schulthess et al., 2017; Sukumaran et al., 2015) covering two main traits, related to grain number and grain dimensions. Various agronomic traits have contributed to grain yield improvement in the past such as plant height, harvest index (HI), total biomass, number of productive tillers, spike length, grain number per spike, and per area and thousand grain weight (TGW). From these, HI and grain number per spike and per area are most important (Foulkes et al., 2011; Philipp et al., 2018). Multiple interactions and compensatory mechanisms exist among the different yield components, depending on genotype × environment interactions (Slafer et al., 2014).

TGWs are one component of yield and thus a potential target for wheat yield improvement (Brinton & Uauy, 2019; Foulkes et al., 2011; Mohler et al., 2016; Tshikunde et al., 2019). Genes involved in the ubiquitin pathway are central regulators of grain size for a number of plants (reviewed by Li & Li, 2014). Ubiquitin covalently binds to target proteins and triggers their degradation in the 26S proteasome complex (Vierstra, 2003). Notably, the ubiquitin proteasome pathway promotes irreversible proteolysis of a set of regulatory proteins absolutely required for cell‐cycle phase transitions (Genschik et al., 2014).

*DA1* encodes an ubiquitin receptor containing two ubiquitin‐interacting motifs (UIMs) and one zinc‐binding LIM domain. DA1 binds polyubiquitinated proteins mediating their degradation by the 26S proteasome (Verma et al., 2004). Arabidopsis *da1*‐*1* was isolated from a genetic screen by showing increased seed and organ size and by producing larger and heavier seeds resulting from enlarged sporophytic integuments (Li et al., 2008). Thus, the size of the maternal outer layers can determine final grain size by setting a physical limit on the available space for the growing filial seed organ (Adamski et al., 2009; Hasan et al., 2011).

In spring wheat, the TaDA1 homeologs reveal high sequence similarities to AtDA1, especially in the UIMs and LIM domains, implying similar protein structures and functions and conserved interaction in the ubiquitin‐proteasome pathway in plants (Liu et al., 2020). While *TaDA1*‐*B* and *TaDA1*‐*D* were predominantly expressed in the vegetative organs such as leaves and roots, TaDA1‐A was principally expressed in young spikes prior to anthesis. DA1‐overexpressing wheat plants contained fewer cells in the outer pericarp. On the other hand, *DA1*‐RNAi plants had more outer pericarp cells, produced a wider pericarp cell layer and increased TGW by around 10%. This confirms that TaDA1 functions to restrict early maternal cell proliferation (Liu et al., 2020). However, it remains unclear whether this increased TGW leads also to an increase in sink capacity and whether it can be translated into a higher wheat grain yield. Increases in grain weight have been frequently reported to have little impact on wheat grain yield often due to the trade‐off between grain weight and grain number (Brinton et al., 2017; Philipp et al., 2018; Song et al., 2007; Wang et al., 2018). However, in some cases, grain yield improvement in wheat has been significantly associated with increased TGW (Brinton & Uauy, 2019; Tshikunde et al., 2019). At least for the *DA1*‐RNAi plants, Liu et al., (2020) reported that the increased grain size was not accompanied by a different grain number per spike.

The positive effect of *da1* on seed size, as shown in wheat and Arabidopsis plants, may provide approaches to improve seed yield. In addition, higher grain size in wheat can positively affect grain composition, flour extraction and/or quality (Nuttall et al., 2017; Wiersma et al., 2001).

Higher grain size and potentially improved sink strength can be relevant to the response to elevated atmospheric CO_2_ concentration (eCO_2_). This is especially important, given the fact that due to anthropogenic activities, the atmospheric CO_2_ concentration is predicted to increase to 550 ppm by 2050 with profound consequences for crop growth (Lemonnier & Ainsworth, 2018; Uddling et al., 2018). While eCO_2_ frequently improves carbon assimilation and increases plant biomass in many species (Taub et al., 2008), sink limitation often occurs, leading to photosynthetic feed‐back inhibition (White et al., 2016). eCO_2_ increases photosynthesis if the sinks are also stimulated (Ainsworth et al., 2004; Aranjuelo et al., 2013). Grain yield in wheat is predominantly sink‐limited during most of the grain filling period when grains grow under saturated source supply (Borras et al., 2004; Sofield et al., 1977). As *da1* increases grain size, we hypothesize that sink limitation could be avoided and that the stimulated photosynthesis could have an improved yield response to eCO_2_ compared to the wild type. Thus, a more efficient photosynthesis under eCO_2_ conditions could be possible at less‐limiting sink capacity and increasing the grain sink strength could be promising to use additional CO_2_ (Wang et al., 2013).

It is therefore hypothesized that grain yield in the *da1* mutant could potentially benefit from additional source stimulation by eCO_2_ when compared to ambient conditions. Wheat genotypes with increased grain size, as achieved in the *da1* mutant, exhibit higher sink capacity/strength at the level of the individual grains. eCO_2_ stimulates source activity, which in many plants stimulates photosynthesis and induces faster growth and biomass accumulation (Amthor, 2001; Jablonski et al., 2002).

In this study, the *da1* wheat mutant was characterized, which provides a suitable model to better understand intrinsic yield determinants in wheat. First, we asked whether the increased TGW in *da1* can be translated into higher wheat grain yield and/or higher sink size and what is the relationship between the yield‐related factors. Second, we wanted to find out whether the additional carbon provided by eCO_2_ can be better used by the *da1* mutant displaying higher sink strength at the level of individual grains. Using such an approach, could simultaneously combine increases of both source (eCO_2_) and sink strength (*da1*). To this end, yield‐related and biomass traits, grain dimensions, and grain quality traits were collected and analyzed in a two‐factorial mixed‐model analysis, regarding genotype and eCO_2_ treatment.

## 2 MATERIAL AND METHODS

### 2.1 Generation of the *da1* mutant

The *da1* mutant derived from an ethyl methanesulfonate‐mutant population established in spring wheat (*Triticum aestivum*, variety Trappe) by KeyGene (www.keygene.com). The mutant population has been screened for mutants in the *DA1* gene. Positive lines have been back‐crossed (BC3) with Trappe to reduce background mutations. The mutations in the different A, B, and D genomes have been combined by crossing.

The *da1* wheat mutant, under analysis in this study, possesses mutations in alleles of the wheat genomes A, B, and D, and in all three cases, the mutation affects the DA1‐domain either by blocking its transcription or by amino acid exchange (Figure S1).

### 2.2 Plant growth

To simulate field‐related conditions, plants were grown under semicontrolled conditions in four small greenhouses (6.1 × 3.4 m) in soil beds with regular irrigation and without supplemental light or temperature regulation for the duration of the experiment (Saalbach et al., 2014). The gables of the greenhouses consist of meshes to ensure optimal ventilation. Outside and inside temperature was recorded throughout the experiment (Figure S2). Grains of *da1* and Trappe were sown on April 3, 2018 and harvested at full maturity at July 18, 2018 on the IPK campus, Saxony‐Anhalt, Germany. A randomized block design was used with six blocks (=replications) per greenhouse in twofold repetition (two greenhouses for both ambient and eCO_2_). Experimental plots (0.5 × 1 m) consisted of four rows (= 160 seeds, which resulted in a density of 360 grains per m^2^) Soil N content was determined and adjusted with fertilization to 180 kg N ha^−1^ before the experiment with no further fertilization during the experiment. eCO_2_ treatment was started at the one‐leaf stage in two greenhouses and continued until physiological maturity, with daily exposition from 5.00 a.m. to 10.00 p.m. During that time, CO_2_ was continuously supplied from gas cylinders via a pipe system surrounding the interior of the respective greenhouses. Levels within the greenhouses were recorded and adjusted to 600 ppm using two CO_2_ sensors per greenhouse. The other two greenhouses served as an ambient control.

### 2.3 Experimental design and data analysis

A two‐factorial experiment was performed. The factors were (i) spring wheat (*Triticum aestivum* L. cv. Trappe) versus the *da1* mutant and (ii) ambient (approx. 410 ppm) versus eCO_2_ (600 ppm). In order to avoid border effects at the front and the back of the plots, all samplings and measurements were performed only for the interior of the two inner rows (30 plants of each inner row = 60 plants per plot), resulting in a single measurement value per plot. Mature spikes from six main tillers per plot were harvested from both *da1* and Trappe under ambient and eCO_2_ (6 spikes × 6 blocks × 2 greenhouses ambient × 2 greenhouses eCO_2_ × 2 genotypes (*da1*, Trappe) = in total 288 spikes) and were harvested and investigated for the total number of spikelets per spike. For spike traits, means were calculated from the harvested spikes of each of the six plots, which were then used for subsequent analysis. HI was calculated from each plot separately. The total dry weights of the 60 harvested plants of the inner rows per plot were determined and divided by the respective total grain weights.

The experiment allowed to study the effect of each factor on response variables related to plant dimensions, yield‐related traits, biomass traits, grain composition, and spike‐related traits. The data were analyzed by mixed‐model ANOVA using OriginPro 8.1 software (www.originlab.com/) and the statistical software program R (www.r‐project.org/).

To verify the results, the experiment with *da1* and Trappe was repeated in 2019 under the same management conditions with eight plots of each genotype. However, the eCO_2_ treatment was omitted in the 2019 experiments.

### 2.4 Analysis of grain morphology, sucrose, starch, carbon, nitrogen, and microelements

Grain dimensions, TGW, grain width, length and area were determined on mature dry grains using the digital seed analyzer MARVIN (www.marvitech.de). The traits HI, grain yield per spike, grain yield per spikelet, and grain number per spikelet were calculated.

From each plot, a sample of mature grains (app. 50 g) was ground by ball‐mill to a fine flour, which was used for subsequent analysis of grain components, macro‐ and microelements. Starch and sucrose contents were determined using a coupled enzyme assay as described (Weigelt et al., 2009). Total carbon and nitrogen in dried wheat flour were determined with the Vario EL Elemental analyzer (www.elementar.de).

Milled flour from multiple mature grains (see above) were weighed into PTFE digestion tubes and digested in HNO_3_ under pressure using a microwave digester (UltraCLAVE IV; MLS). Macroelement and microelement were measured by inductively coupled plasma optical emission spectrometry (ICP‐OES, iCAP 6500, Thermo Fisher Scientific) combined with the CETAC ASXPRESS™ PLUS rapid sample introduction system, and a CETAC autosampler (CETAC Technologies). Element standards were prepared from certified reference materials from CPI international (Eroglu et al., 2017).

## 3 RESULTS

### 3.1 Sequences alignment and phylogenetic analysis

DA1 from the genome B was selected to perform a blastp search of orthologs on EnsemblPlants (https://plants.ensembl.org/index.html). Those sequences with a percentage of identity higher than 60% were selected for the following analyses, i.e., *Zea mays* Zm00001d035844_T012, *Hordeum vulgare* HORVU2Hr1G002700.14, *Sorghum bicolor* SORBI_3010G064600:KXG19479, *Triticum dicoccoides* TRIDC2AG001610.3, *Brachypodium distachyon* BRADI_4g42580v3:KQJ92266, *Glycine max* GLYMA_17G247700:KRH05774, *Triticum turgidum* TRITD2Av1G003900.3, *Oryza sativa Japonica group* Os06t0182500‐02, Arabidopsis thaliana AT1G19270.1, Aegilops tauschii AET0Gv20035900.10, *Triticum urartu* TRIUR3_12237:TRIUR3_12237‐T1, *Medicago truncatula* MTR_5g018900:AES94835, *Manihot esculenta* MANES_10G022200:OAY38533, *Cucumis sativus* Csa_2G286500:KGN62017, and *Beta vulgaris* BVRB_6g138060:KMT08523). Amino acid alignment was performed with CLUSTALW followed by the construction of an unrooted phylogenetic tree using MEGA‐X v10.1.5 with the ML (Maximum Likelihood) and NNI (Nearest‐Neighbour‐Interchange) JTT model on a 2000 bootstrap method (Kumar et al., 2018).

The ClustalO alignment indicated that the sequences TraesCSU02G00780.1, TraesCS2B02G007700.1 and TraesCS2D02G0169.1 correspond to DA1 genes of the genome A, B and D respectively and share 99% identity (Figure S3). The DA1 gene in wheat encodes 504 amino acids and harbors a DA1‐typical domain, ubiquitin interacting motif domain (UIM), and LIM‐type zinc finger domain (Znf_LIM), (Figure S4). Wheat DA1 has high identity to the homeologs of barley (98%), rice (88%), maize (86%), millet (86%), and Arabidopsis (60%), which suggests similar functions across plant species.

### 3.2 Plant performance and grain dimensions

After sowing, the germination rate was determined as 99%–100% with no apparent genotype and treatment effects. Days to anthesis (app. at 61 days after sowing) and length of flowering (app. 3.5 days) were found to be not dependent on either genotype or treatment.

**FIGURE 1:**
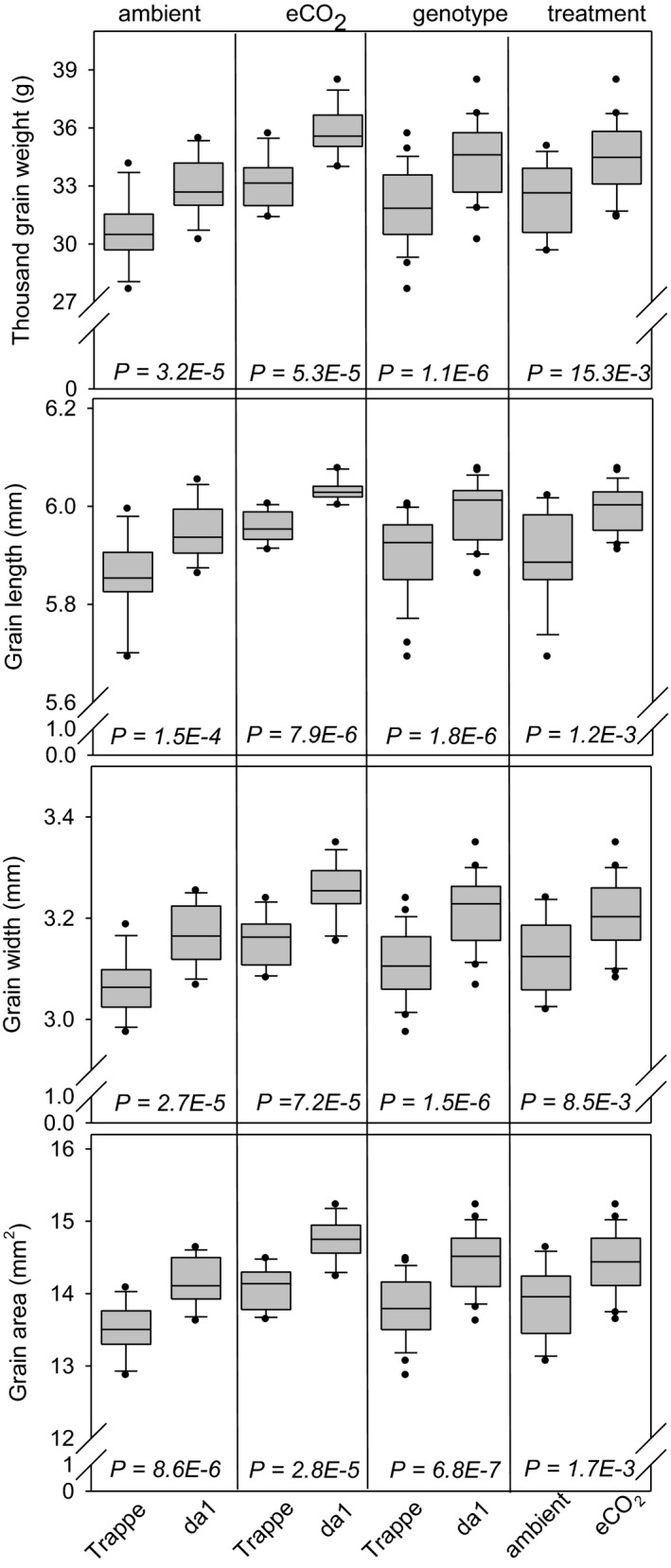
Distribution of grain dimension‐related traits represented by box and whisker plots, measured for *da1* wheat and wild‐type Trappe. The boundary of the box closest to zero indicates the 25th percentile, the line within the box marks the median, and the boundary of the box farthest from zero indicates the 75th percentile. Whiskers (error bars) above and below the box indicate the 90th and 10th percentiles. Plots show all data points that lie outside the 10th and 90th percentiles. The 4 inserts from left to right show the influence of ambient CO_2_, eCO_2_, genotype, and treatment for Trappe (left) and *da1* (right). *p* values indicate statistical significance between values. Two factors, genotype, and treatment influencing grain dimension traits

The analysis of grain dimensions of mature grains revealed that TGW for *da1* was increased by 8% (*p* = 3.2E‐5). eCO_2_ further enlarged TGW for *da1* and Trappe, each by another approx. 8% (*p* = 5.3E‐5, Figure 1). The increased TGW by both genotype and treatment was reflected by parallel and highly significant increases of grain length, width and area (Figure 1). Thus, the *da1* mutant exhibited increased TGW compared to Trappe, and eCO_2_ further increased TGW in a similar manner in both genotypes.

### 3.3 Yield‐related traits

**FIGURE 2:**
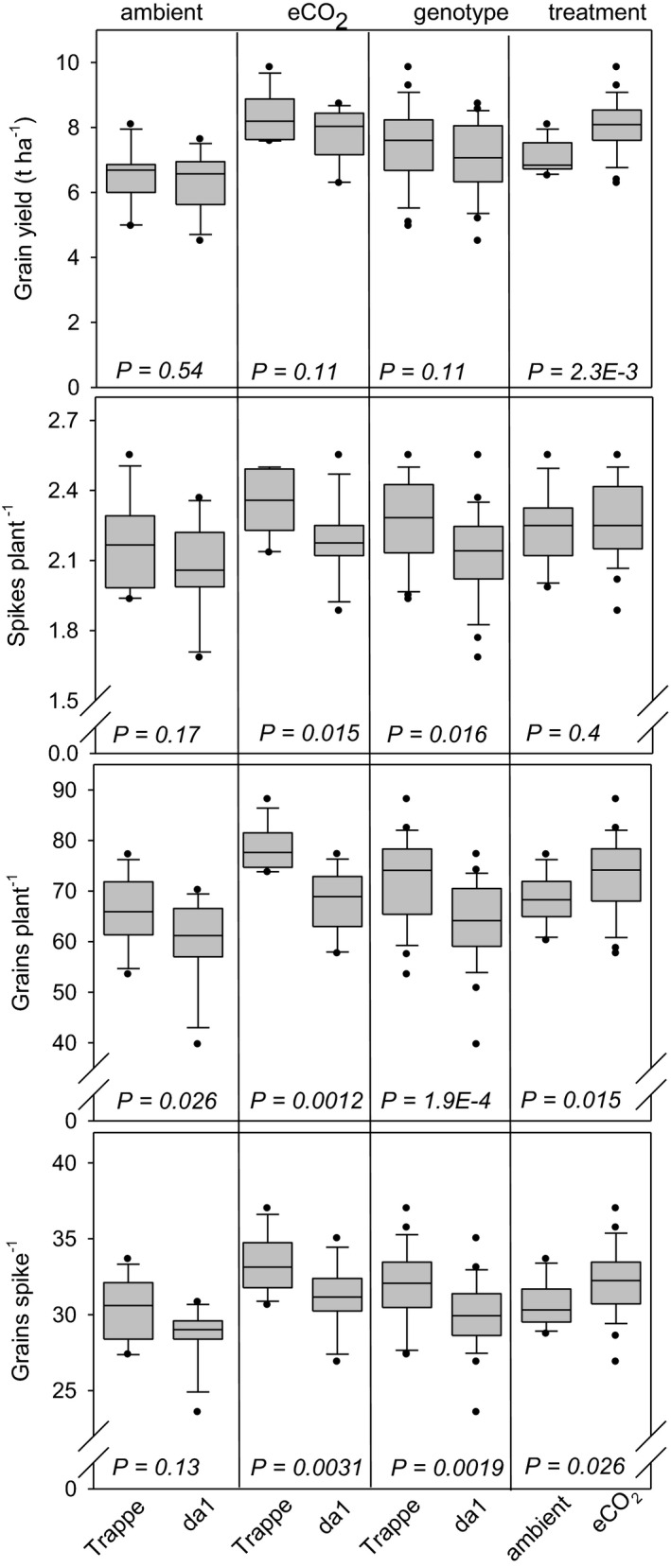
Distribution of yield‐related traits represented by box and whisker plots, measured for da1 wheat and wild‐type Trappe. The boundary of the box closest to zero indicates the 25th percentile, the line within the box marks the median, and the boundary of the box farthest from zero indicates the 75th percentile. Whiskers (error bars) above and below the box indicate the 90th and 10th percentiles. Plots show all data points that lie outside the 10th and 90th percentiles. The four inserts from left to right show the influence of ambient CO_2_, eCO_2_, genotype and treatment for Trappe (left) and *da1* (right). *p* values indicate statistical significance between values. Two factors, genotype and treatment, influence grain dimension traits

Grain yield calculated as tons/ha was not different between *da1* and Trappe but was significantly increased by 27% and 25% upon eCO_2_ treatment for *da1* and Trappe, respectively (Figure 2). Spike number per plant was unchanged by eCO_2_ treatment but the genotype effect revealed 6% more spikes per plant for Trappe compared to *da1*.

While grain number per plant was significantly enhanced under eCO_2_ by 19% and 13% for Trappe and *da1* respectively, there was a negative effect of the genotype resulting in 10% and 15% lower grain number per plant for *da1* under ambient and eCO_2_ conditions compared to Trappe, respectively. Similarly, the trait grain number per spike increased upon eCO_2_ in both Trappe and *da1* by 9% and 8%, respectively, whereas the genotype effect decreased grains per spike in *da1* by app. 6% under ambient and eCO_2_ conditions. Taken together, the *da1* mutant displayed reduced spikes per plant, grains per plant and grains per spike, independent of the treatment, which could outcompete the higher TGW (Figure 1), resulting in no gain of grain yield per area of *da1* compared to Trappe.

### 3.4 Biomass traits

**FIGURE 3:**
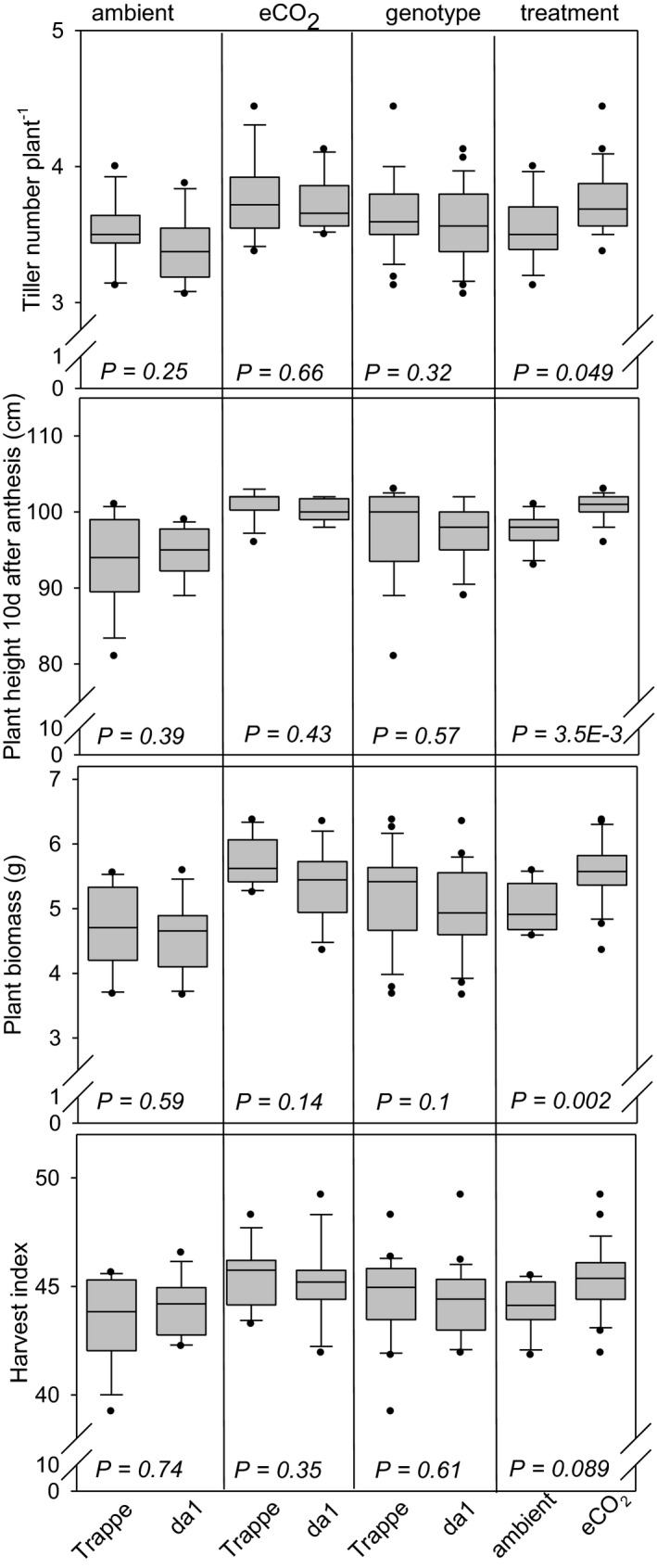
Distribution of biomass traits represented by box and whisker plots, measured for da1 wheat and wild‐type Trappe. The boundary of the box closest to zero indicates the 25th percentile, the line within the box marks the median, and the boundary of the box farthest from zero indicates the 75th percentile. Whiskers (error bars) above and below the box indicate the 90th and 10th percentiles. Plots show all data points that lie outside the 10th and 90th percentiles. The four inserts from left to right show the influence of ambient CO_2_, eCO_2_, genotype, and treatment for Trappe (left) and *da1* (right). *p* values indicate statistical significance between values. Two factors, genotype and treatment, influence grain dimension traits

eCO_2_ in many plants stimulates source activity, photosynthesis and induces faster growth and biomass accumulation (Jablonski et al., 2002). On the other hand, DA1 is supposed to be a regulator of cell proliferation in maternal seed organs (Liu et al., 2020). Therefore, possible effects of eCO_2_ treatment and genotype on biomass traits were analyzed (Figure 3). Tiller number at the beginning of the stem elongation stage was not different between the genotypes but increased similarly by 7% and 9% upon eCO_2_ for Trappe and *da1*, respectively. Whereas plant height at anthesis was not different between genotypes at either treatment (data not shown), eCO_2_ increased plant height at 10 days after anthesis by 6% and 7% for Trappe and *da1*, respectively, compared to ambient CO_2_. Plant biomass was independent of the genotype but increased upon eCO_2_ by 22% and 17% for Trappe and *da1*, respectively. HI did not change by genotype but was slightly higher upon eCO_2_ treatment by 4% for both *da1* and Trappe. Interactions between the two factors, eCO_2_ and genotype, were calculated for all combinations but were not significant.

Taken together, the results revealed no differences in the biomass traits analyzed between *da1* and Trappe. As expected, eCO_2_ treatment leads to an increase in these traits, with no apparent differences in the response between *da1* and Trappe.

### 3.5 Spike‐related traits

**FIGURE 4:**
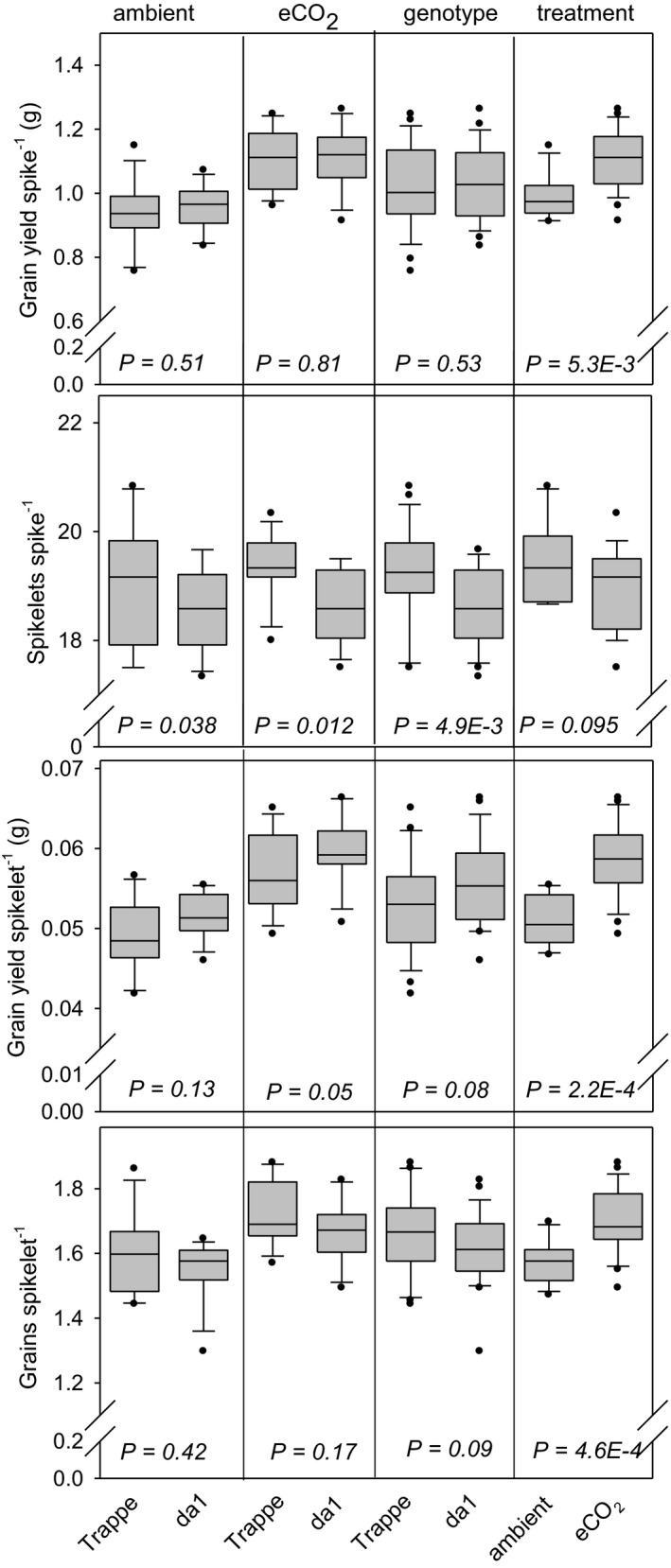
Distribution of spike‐related traits represented by box and whisker plots, measured for da1 wheat and wild‐type Trappe. The boundary of the box closest to zero indicates the 25th percentile, the line within the box marks the median, and the boundary of the box farthest from zero indicates the 75th percentile. Whiskers (error bars) above and below the box indicate the 90th and 10th percentiles. Plots show all data points that lie outside the 10th and 90th percentiles. The four inserts from left to right show the influence of ambient CO_2_, eCO_2_, genotype, and treatment for Trappe (left) and *da1* (right). *p* values indicate statistical significance between values. Two factors, genotype and treatment, influence grain dimension traits

The results described above indicated specific changes at the level of grain number and grain size. We therefore analyzed spike related traits. The genotype and treatment effects are shown in Figure 4. Grain yield per spike did not differ between *da1* and Trappe for both ambient and eCO_2_ conditions, lacking a genotype effect. However, levels increased significantly upon eCO_2_ treatment by 18% and 16% for *da1* and Trappe, respectively, showing a clear treatment effect. Spikelets per spike did not show significant treatment effects upon eCO_2_, but with a trend to lower levels due to eCO_2_ (not significant at *p* < 0.05). However, the genotype effect led to approx. 3% less spikelets per spike in *da1* compared to Trappe.

Grain yield per spikelet was not changed between *da1* and Trappe for both ambient and eCO_2_ conditions, lacking a genotype effect. Grain number per spikelet behaved similar, lacking a genotype effect. However, both, grain yield per spikelet and grain number per spikelet increased upon eCO_2_ by 15% and 8%, respectively.

Taken together, the results indicated reduced numbers of spikelets per spike in *da1*, which is probably balanced by a higher TGW (Figure 1) resulting in no gain of grain yield per area of *da1* compared to Trappe.

### 3.6 Grain composition

While eCO_2_ can increase grain yield of wheat there is often a shift in grain components and functional properties (Fangmeier et al., 1997; Högy et al., 2009). Likewise, differences in grain size can change the relationship between seed organs affecting grain composition and quality (Nuttall et al., 2017). Therefore, possible effects of treatment and genotype on the composition of mature grains were analyzed.

**FIGURE 5:**
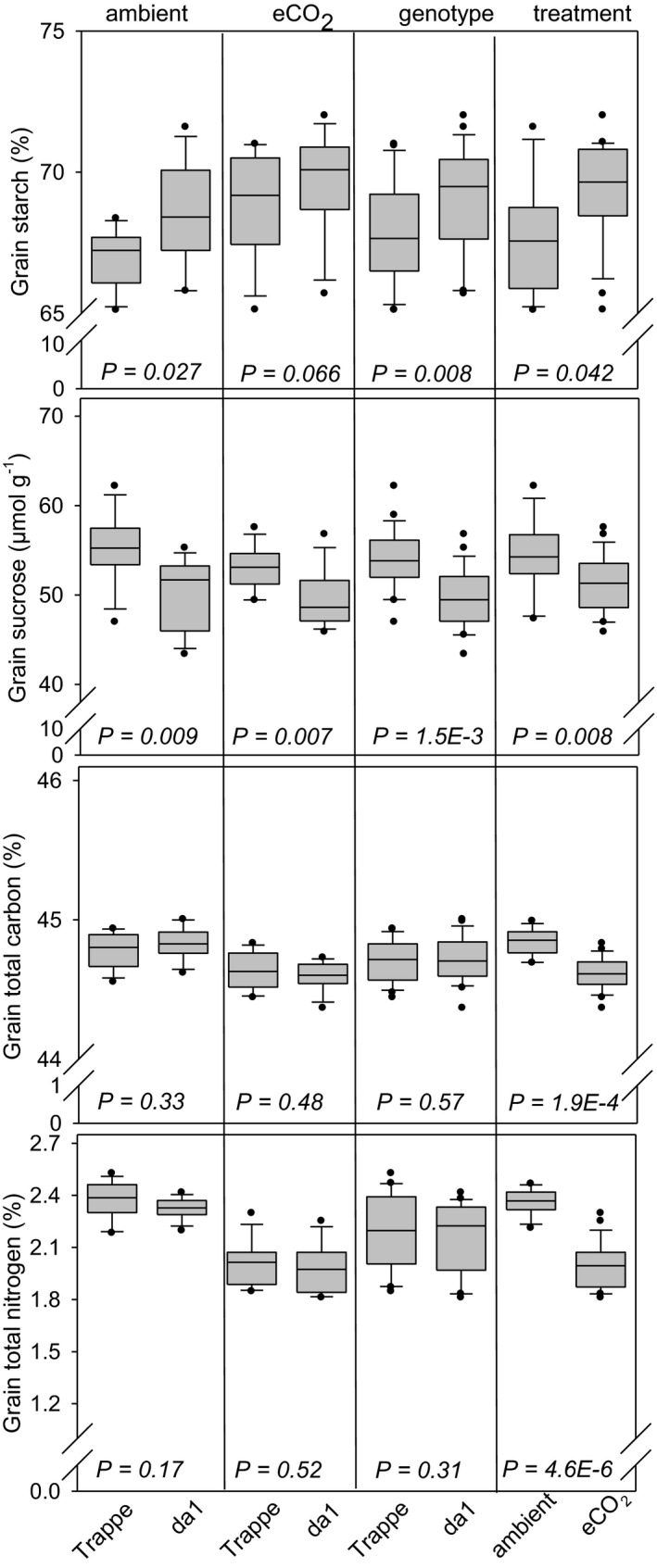
Distribution of grain composition represented by box and whisker plots, measured for da1 wheat and wild‐type Trappe. The boundary of the box closest to zero indicates the 25th percentile, the line within the box marks the median, and the boundary of the box farthest from zero indicates the 75th percentile. Whiskers (error bars) above and below the box indicate the 90th and 10th percentiles. Plots show all data points that lie outside the 10th and 90th percentiles. The four inserts from left to right show the influence of ambient CO_2_, eCO_2_, genotype and treatment for Trappe (left) and *da1* (right). *p* values indicate statistical significance between values. Two factors, genotype and treatment, influence grain dimension traits

Grain starch content was increased by approx. 2% in *da1* grains compared to Trappe under ambient conditions and by 3% in response to eCO_2_. Sucrose content in mature grains was 9% lower for *da1* compared to Trappe under ambient conditions and 6% under eCO_2_. The results showed that both *da1* effects and eCO_2_ only slightly affected starch and sucrose levels. Both factors resulted in a small increase of the starch to sucrose ratio. Grain total carbon content of mature grains was unchanged between *da1* and Trappe but was slightly lower upon eCO_2_ treatment, however with only 0.5%. Grain total nitrogen content was not different between *da1* and Trappe but decreased significantly upon eCO_2_ treatment by as much as 15% in both *da1* and Trappe (Figure 5).

The concentrations of major macro‐ and microelements were measured by ICP‐OES (Table 1). The analysis of flour from mature grains revealed that *da1* did not differ considerably from Trappe with respect to important grain nutrient and microelement concentrations including Fe, Zn, S, Mn, Mg, K, Ca, Na, and N. Significant genotype‐dependent differences were found only for Na with 8% lower values in *da1*. In contrast, eCO_2_ treatment strongly decreased the concentrations of several of the essential elements, including Fe, Zn, S, Mn, and N by 7%, 12%, 12%, 10%, and 15%, respectively, whereas levels of Mn, K, Ca, and Na were unchanged.

**TABLE 1:** Grain macro‐ and micro elements

µg/g	Ambient CO_2_					eCO_2_					Genotype					eCO_2_ treatment					
	Trappe		*da1*			Trappe		*da1*			Trappe		*da1*			ambient CO_2_		eCO_2_			
	MW	SD	MW	SD	*p*	MW	SD	MW	SD	*p*	MW	SD	MW	SD	*p*	MW	SD	MW	SD	*p*	
Fe	43.57	3.33	43.51	4.45	*0.97*	38.81	3.61	39.70	4.22	*0.5*	41.19	4.18	41.69	4.68	*0.4.4*	42.15	2.48	39.09	3.64	***0.01***	
Zn	41.63	2.98	42.04	5.23	*0.82*	35.51	3.32	36.73	4.53	*0.44*	38.57	4.39	39.39	5.50	*0.3*	41.24	3.77	36.12	3.93	***0.003***	
S	1849.34	84.90	1843.99	108.94	*0.89*	1613.93	125.97	1611.98	135.33	*0.96*	1731.64	159.66	1727.99	168.75	*0.71*	1824.37	79.84	1612.96	127.86	***0.000***	
Mn	43.40	5.19	42.98	4.20	*0.84*	37.18	3.70	37.54	3.50	*0.8*	40.29	5.43	40.26	4.69	*0.49*	41.50	3.93	37.36	3.52	***0.011***	
Mg	1461.40	115.42	1481.99	139.07	*0.72*	1334.60	106.99	1402.97	121.90	*0.22*	1398.00	126.65	1442.48	134.11	*0.12*	1423.46	100.45	1368.79	117.48	*0.17*	
K	6546.57	397.98	6472.35	564.25	*0.76*	6566.76	400.75	6564.18	562.71	*0.99*	6556.66	390.73	6518.27	553.09	*0.8*	6497.41	410.34	6565.47	477.75	*0.68*	
Ca	618.97	90.75	623.27	80.29	*0.900*	540.57	55.65	564.93	50.04	*0.270*	579.77	83.81	594.10	71.89	*0.530*	573.30	63.65	552.75	53.23	*0.310*	
Na	12.78	1.33	11.64	1.32	*0.088*	12.92	1.40	12.02	1.10	*0.36*	12.85	1.33	11.83	1.21	*0.13*	11.76	0.97	12.47	1.31	*0.12*	
N	2373.72	105.18	2328.41	58.44	*0.17*	2008.45	124.99	1980.55	136.19	*0.52*	2191.08	218.10	2154.48	205.11	*0.31*	2360.71	71.24	1994.50	128.63	***0.000***	

Note

*n* = 24, *p* values in bold, significant differences according to mixed‐model ANOVA, *p* < 0.05.

Factors genotype and eCO_2_ treatment influencing the concentration of nutrient elements in mature grains of *da1* and Trappe.

### 3.7 Validation on ambient conditions

In order to confirm the results from the 2018 experiment, the field trial was repeated in the following year 2019 with *da1* and Trappe, while the eCO_2_ treatment was omitted. Figure S5 shows the data as a comparison between *da1* and Trappe and between the 2 years. According to the first‐year's results, *da1* again exhibited significantly increased TGW, grain length, width, and area. Similar to the 2018 experiment, grain yield and spike number per plant were not different between *da1* and Trappe. Thus, the trial in 2019 confirmed the differences between *da1* and Trappe. Grain morphology traits such as TGW, grain length, area, and width behaved similarly as in the 2018 trial, thus confirming the increased grain size phenotype of *da1* mutants. Likewise, yield parameter results were consistent between both experiments; grain yield per spike was not different between both lines. The *da1* mutant exhibited increased grain size but decreased grain number per plant and spike.

## 4 DISCUSSION

Grain size in wheat is a potential target to improve yield potential. The ubiquitin pathway affects organ growth and seed size, which is negatively regulated by the ubiquitin receptor DA1 restricting early maternal cell proliferation in Arabidopsis and crop plants such as canola, maize and wheat (Li & Li, 2014; Liu et al., 2020; Wang et al., 2017; Xie et al., 2018). The *da1* wheat mutant increases grain size and provides a suitable model to better understand intrinsic yield determinants in wheat. The aim was to study whether increased grain size in da1 can be translated into higher wheat grain yield and/or higher sink size and what is the relationship between the yield‐related factors in *da1*. In order to alleviate possible source limitation, it was also tested whether the additional carbon provided by high CO_2_, expected in the future, can be better used by the *da1* mutant.

### 4.1 The *da1* mutation and elevated CO_2_ independently increase grain size

The *da1* wheat mutant exhibited 8% increased TGW compared to Trappe (Figure 1). This increase is related to both grain length and width and indicated higher sink strength of individual grains. Accordingly, the Arabidopsis *da1*‐*1* mutant produces larger and heavier seeds resulting from enlarged sporophytic integuments (Li et al., 2008). In wheat, grain size is associated with carpel size (Calderini et al., 1999) and with variations in the ovary wall size, which is related to cell number rather than cell size (Reale et al., 2017). Furthermore, grain length and pericarp cell length are associated (Muñoz & Calderini, 2015; Pielot et al., 2015). TaDA1 has been described as a negative regulator of grain size. *DA1*‐RNAi plants resembles the wheat *da1* mutant and produced more outer pericarp cells at 15 DAP, formed a wider pericarp cell layer, and displayed increased grain size by around 10% (Liu et al., 2020). It was also shown that TaDA1‐A is predominantly expressed in young spikes at pre‐anthesis (Liu et al., 2020). Cell division in the cereal pericarp is terminated as early as 2 days after fertilization (Radchuk et al., 2011). Thus, it is hypothesised that the impact of *da1* on grain size most probably comes from a prolonged cell division phase in the early maternal grain tissue, probably before anthesis. Hence, the increased maternal grain layers may overcome physical barriers to endosperm expansion, thereby increasing the available space for endosperm growth, which finally leads to increased grain size (Hasan et al., 2011).

Our results reveal that eCO_2_ further increased TGW in a similar manner for both genotypes by app. 8%, independently from the *da1*‐effects (Figure 1). In plants, eCO_2 _has been shown to enhance cell division, to shorten the duration of the cell cycle, and to promote cell production as well as expansion (Gamage et al., 2018). Thus, eCO_2_ could possibly further stimulate the cell proliferation in the developing grains. This is probably due to the eCO_2_‐mediated carbon “fertilization effect,” which mediates an assimilate and/or sugar supply effect stimulating cell proliferation within the developing grain (Lastdrager et al., 2014; Weichert et al., 2017).

### 4.2 Improvement of grain size in *da1* is compensated by lower grain number

The finding that biomass traits such as tiller number, plant height at 10 days after anthesis, HI, and spike number per plant were not changed in *da1* indicates that the mutation does not change vegetative parameters but preferentially affects grain development around anthesis. While grain size was increased in *da1* compared to Trappe (+8%, Figure 1), some of the other important yield components were decreased (Figure 2) such as spikes per plant (−6%), grains per plant (−12%), and grains per spike (−6%). However, total grain yield was not altered (Figure 2). These results reveal that the improvement of grain size is counter balanced in a way that final grain yield is not altered. Thereby, the larger grain size was compensated by several other traits related to grain number per spike such as less spikes per plant, lower grain number per spike, lower number of spikelets per spike and, eventually less grains per spikelets, even the latter was not significant here (Figure 4). Altogether, this results in a lower number of grains per plant and per area in *da1* compared to Trappe. The comparison between *da1* and Trappe confirms the well‐known trade‐off between grain size and grain number, which finally maintains grain yield stability (Sadras, 2007).

### 4.3 The *da1* mutation and elevated CO_2_ additively increase grain size but not grain yield

Both *da1* and eCO_2_ independently stimulate sink activity on the level of the individual grain. However, it can be hypothesised that *da1* acts only on early grain development by sustaining cell proliferation without directly affecting source strength or assimilate availability. This may cause competition for assimilates between individual grains within the spike, resulting in the compensation of the larger grain size by less grains per plant and per spike. In contrast, eCO_2_ operates at the whole plant level including grain development by a metabolite/assimilate effect, which becomes evident by a stimulation of different biomass traits (Figure 3) such as higher grain number per plant (+7%), grain yield (+14%, Figure 2) and grain number per spikelet (+8%, Figure 5). Additional supply of eCO_2_ produced a significant increase of grain size in Trappe and *da1* each by 8% (Figure 1), indicating that TGW in both genotypes can benefit in a similar manner from eCO_2_. Thus, the CO_2_ treatment and the *da1* mutation increased grain size additively leading to an increase in TGW of 17% in the *da1* × eCO_2_ combination compared to Trappe at ambient CO_2_. However, the reduction of grain number is still maintained in the *da1* × eCO_2_ combination compared to Trappe × eCO_2_.

Although the da1 mutation increased sink strength at the level of the individual grain, sink size at the whole plant level is not changed, due to a trade‐off between grain size and grain number and evidenced by unchanged grain yield. It has been shown that sink size is highly relevant for the eCO_2_ response (Wang et al., 2013). In durum wheat, the inability to develop corresponding sinks leads to photosynthetic acclimation, which constrained any eCO_2_ effects on yield (Aranjuelo et al., 2013). The failure of the *da1* mutation to increase sink size on the whole plant level could therefore explain the fact that eCO_2_ does not benefit grain yield of *da1* superior to the wild type. Thus, it can be argued that total yield in the genetic background of a *da1* mutation cannot profit from source stimulation by higher CO_2_, expected in the future due to climate change, as final yield gain remained unchanged between *da1* and Trappe at eCO_2_.

In summary, even though eCO_2_ increases yield and grain number in general, it cannot overcome the negative effect of the *da1* genotype, which goes back to a trade‐off between grain size and grain number and the failure in increase sink capacity. Thus, total yield in the genetic background of a *da1* mutation cannot profit from source stimulation by higher CO_2_, expected in the future due to climate change.

### 4.4 Grain quality is not altered by *da1* but is impaired by elevated CO_2_

Grain starch and sucrose contents were only slightly altered by both *da1* mutation and eCO_2_, leading to a small increase of the starch to sucrose ratio in *da1* (Figure 5). Moreover, the concentration of essential macroelement and microelement in *da1* grains were not changed with respect to Trappe (Table 1). In contrast, eCO_2_ treatment strongly decreased several of the macroelement and microelement similarly in *da1* and Trappe, such as Fe, Zn, S, and Mn by 7%, 12%, 12%, and 10%, respectively. However, other elements remain constant such as Mg, K, Ca, and Na. C3 plants such as wheat generally respond to eCO_2_ with increased photosynthesis, reduced stomatal conductance and a significant reduction in the amount of essential elements in grains (Amthor, 2001; Högy & Fangmeier, 2008; Myers et al., 2014; Pleijel & Högy, 2015). It has been suggested that reduced transpiration‐driven mass flow of nutrients under eCO_2_ contributes to decreases in seed concentrations of several elements (Houshmandfar et al., 2018). Especially the grain N concentration decreased upon eCO_2_ by 15% (Table 1). CO_2_ enrichment causes lower N and protein levels in nonleguminous C3 species and alters acquisition, remobilization, redistribution, and accumulation of N (Taub & Wang, 2008). eCO_2_ physiologically induces N deficiency, reducing both nitrate uptake from soil and nitrate reduction, while ammonium uptake is favored (Bloom et al., 2014).

### 4.5 Enhancing grain size via *da1* is not a suitable way to increase yield potential in wheat

The negative relationship between grain size and grain number is an intrinsic property of many crop and noncrop plants (Acreche & Slafer, 2006; Quintero et al., 2018). From an evolutionary point, the adjustment between these two traits is important and guarantees yield stability (Sadras, 2007). This trade‐off is probably not easy to overcome by conventional breeding and results from the complex interaction of source limitation such as shortage of assimilate supply to grains, sink limitation such as inability of each grain to unload and/or accumulate assimilates and translocation limitation such as inefficient delivery of assimilates from leaves to grains (Seki et al., 2015).

The breeding process in the past essentially increased grain number per area by enhancing grains per spike and spikelets, however, without much gain in TGW (Philipp et al., 2018). An increase of grain number per spike strongly depends on assimilates allocated to the spike (Ghiglione et al., 2008). Competition for assimilates occurs within the spike and is controlled by assimilate loading and unloading within the vascular system and the short‐distance transport within the spike, rachi, and spikelets. Possible limitations in transport capacities and competition for assimilates between spikelets and/or florets could impact biomass distribution among individual tissues within the spike (Reynolds et al., 2009). A potential issue could be to target resistance to assimilate movement imposed by the vascular system of the spike. Resistance to assimilate movement within the spike and particularly that within the spikelets, may be an important component of spike “sink activity” and a possible limitation to yield (Bremner & Rawson, 1978). Disparity in number and dimensions of vascular bundles in different spike segments could be critical affecting ultimate size and grain number along the rachis (Asli & Houshmandfar, 2011). In rice, simultaneous increases in sink size and translocation capacity through the vascular bundles increased the number of vascular bundles and contributed to increased grain yield (Fujita et al., 2013; Terao et al., 2010).

Eventually, genetic yield gain during breeding has not been accompanied by similar increases in the vasculature size of the wheat spike. Accordingly, no clear association was found between the genetic improvement and magnitude of vascular systems in peduncles of the wheat spike (Lopez‐Garrido et al., 2001). Hence, the spike architecture in terms of the relative distribution of grain yield and number along the spike is surprisingly stable and has not been improved by breeding in the past (Philipp et al., 2018).

The results in this study give valuable insights into the interactions among yield components related to grain size and grain number and the possible limitations of yield potential in wheat. Apparently, stimulation of sink strength at the individual grain level by increasing cell proliferation, as achieved in *da1* grains, will not increase total yield, unless the trade‐off between grain size and grain number can be overcome and an increase of sink capacity can be achieved. While eCO_2_ increased yield and grain number additively and independently of *da1*, it did not overcome the trade‐off between grain size and number in *da1*. The attempt to increase grain sink strength by ectopic expression of a sucrose transporter in the wheat endosperm increased individual grain weight but also decreased grain number per spike, thereby confirming the predominant trade‐off between grain size and number (Saalbach et al., 2014; Weichert et al., 2010). This consistent relationship supports the conclusion that the improvement of grain yield is best achieved through an integrated approach targeting several yield‐component traits in parallel (Würschum et al., 2018).

## CONFLICT OF INTEREST

The authors declare that they have no conflict of interest.

## AUTHOR CONTRIBUTION

I.M.‐R., H. Wei., and H.W.: investigation, formal analysis, funding acquisition, and writing – review & editing; H.W.: data curation, project administration, and writing‐original draft; N.v.W.: investigation, writing – review & editing; BASF colleagues, funding acquisition, resources, conceptualization, data curation, and writing – review & editing.

## SUMMARY STATEMENT

The wheat da*1* mutation and elevated CO_2_ additively and independently increase grain size but not yield due to trade‐offs between grain size and number. Elevated CO_2_ but not *da1* impairs grain nutrient and microelements.

## Supporting information

Fig S1‐S5
