# Supplementary material for: The da1 mutation in wheat increases grain size under ambient and elevated CO2 but not grain yield due to trade‐off between grain size and grain number
Source: Plant Environ Interact. 2021 Mar 13;2(2):61–73. doi: 10.1002/pei3.10041 (PMC10168082; doi:10.1002/pei3.10041)
Supplement: Supplementary file 1 — Fig S1‐S5 [file PEI3-2-61-s001.pdf]

Allele	gDNA change	CDS position	AA change	Mutation Type
A	G>A	419	-	Splice site
B	G>A	675	W225*	Stop gained
D	G>A	983	R328K	Non_synonymous

Supplementary Fig. S1, Overview on the location of the mutations in the *da1*-wheat.

Fig. S2, Mean day temperature as recorded by the IPK Gatersleben weather station 2018 (red); mean day temperature as recorded within the greenhouse (black).

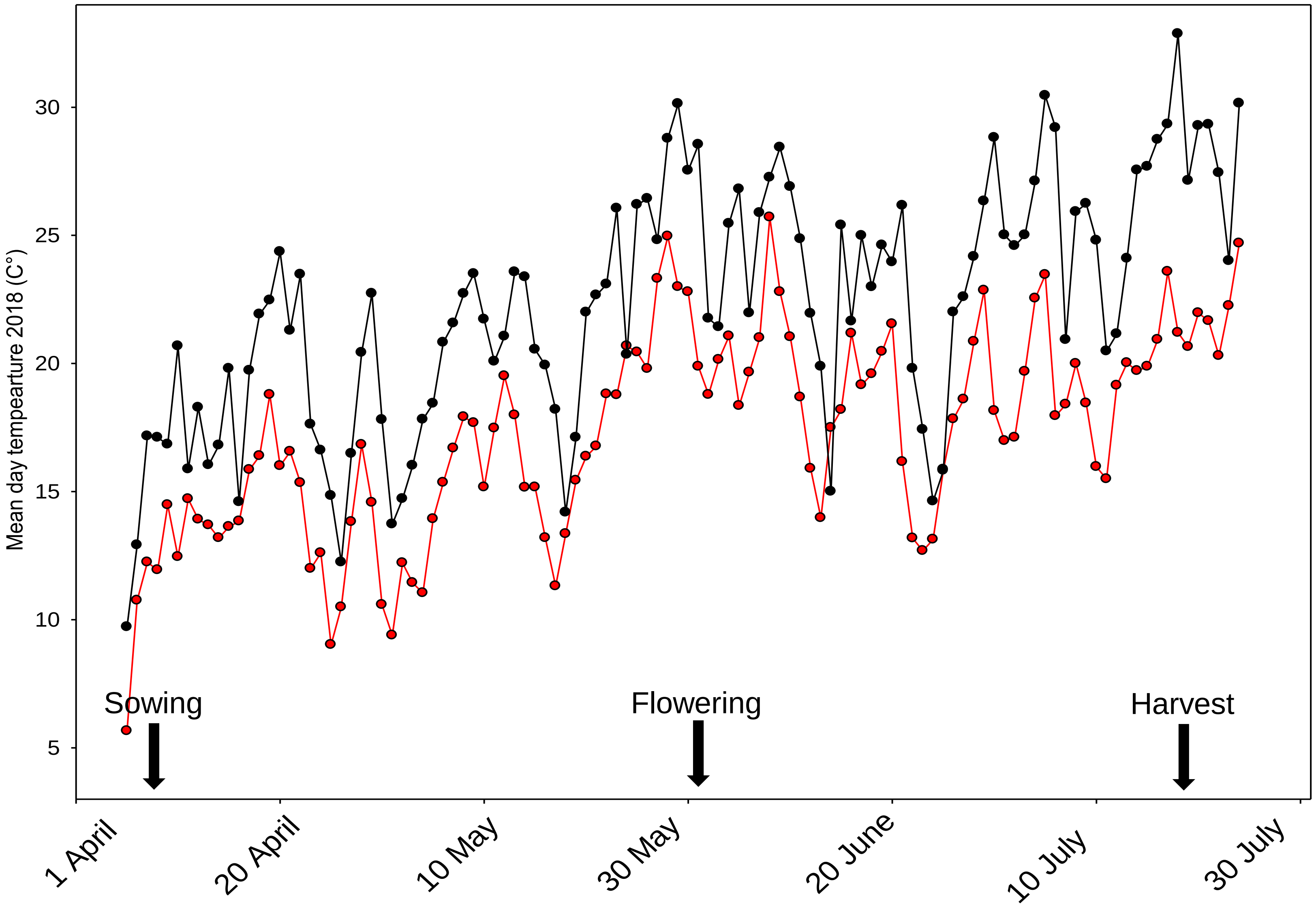

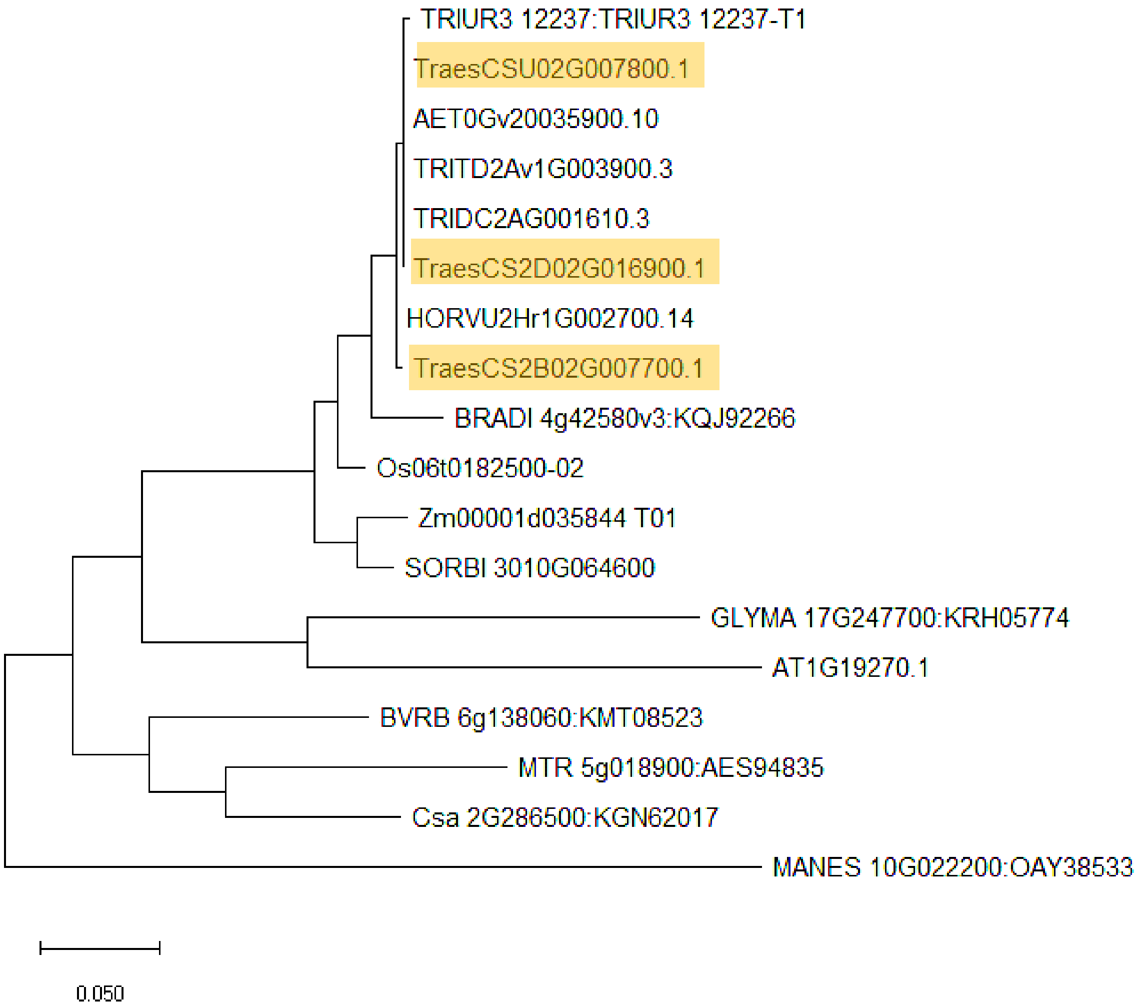

Fig. S3, Phylogenetic tree of selected DA1 proteins.
Wheat DA1 sequences are shaded. *Zea mays* Zm00001d035844_T012, *Hordeum vulgare* HORVU2Hr1G002700.14, *Sorgum bicolor* SORBI_3010G064600:KXG19479, *Triticum dicoccoides* TRIDC2AG001610.3, *Brachypodium distachyon* BRADI_4g42580v3:KQJ92266, *Glycine max* GLYMA_17G247700:KRH05774, *Triticum turgidum* TRITD2Av1G003900.3, *Oryza sativa* Japonica group Os06t0182500-02, *Arabidopsis thaliana* AT1G19270.1, *Aegilops tauschii* AET0Gv20035900.10, *Triticum urartu* TRIUR3_12237:TRIUR3_12237-T1, *Medicago truncatula* MTR_5g018900:AES94835, *Manihot esculenta* MANES_10G022200:OAY38533, *Cucumis sativus* Csa_2G286500:KGN62017 and *Beta vulgaris* BVRB_6g138060:KMT08523.

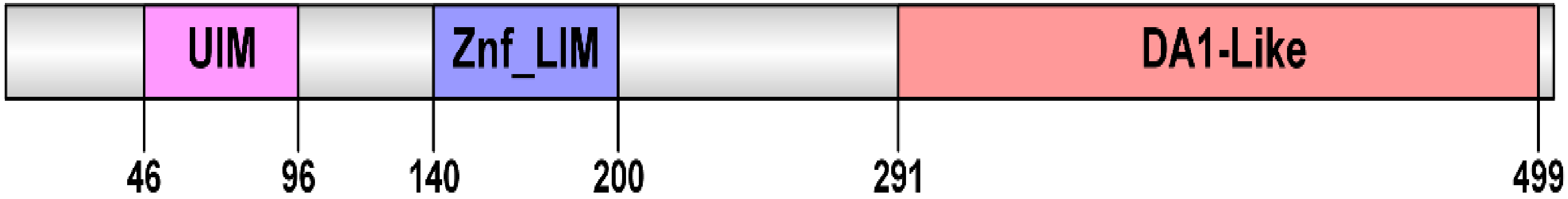

Supplementary Fig. S4, Schematic representation of the DA1 wheat sequence and its domains.

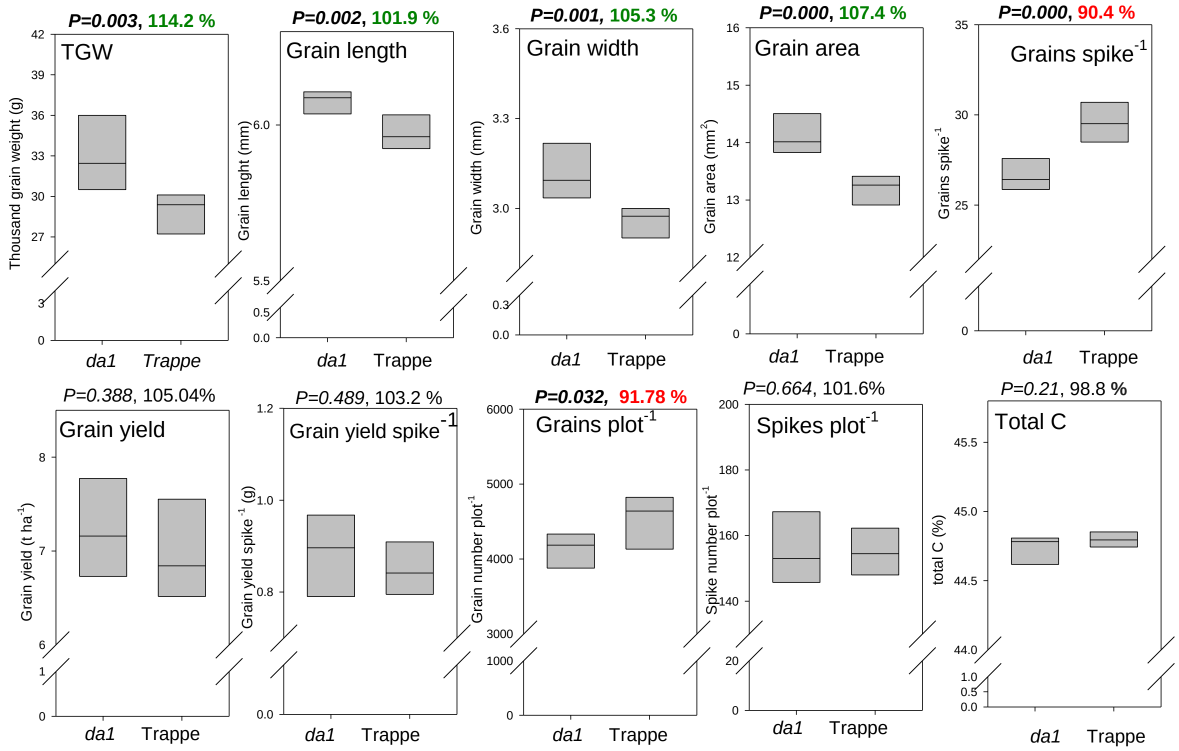

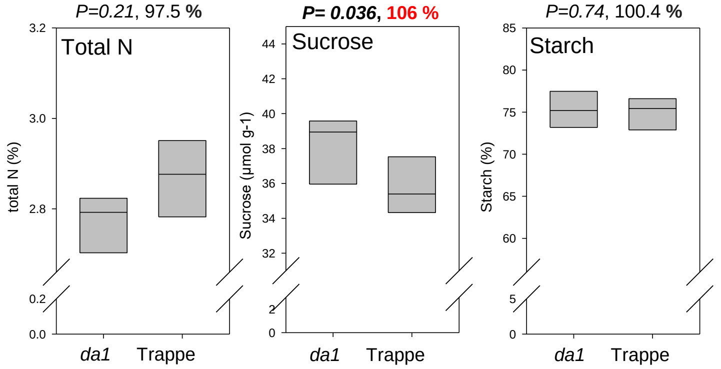

da1 relative to Trappe	2018	2019
Grain yield	98.0	105.0
Spike number per plant	95.2	101.6
Grain area	**105*****	**107.4*****
Grain length	**101.6*****	**101.9****
Grain width	**103.3*****	**105.3****
TGW	**107.9*****	**114.2****
Grain number per spike	**95***	**90.4*****
Grains per plant	**90.5***	**91.8***
Spikelets per spike	**97.2***	nd
Grain yield per spike	102.2	103.2
C	100.1	99.8
N	98.1	97.5
sucrose	90.5	**106.2***
starch	102.3	100.4

Fig. S5, Repetition experiment. Box plots show the distribution of grain dimension-, yield-, biomass-, and grain composition-related traits, measured for *da1* wheat and wildtype Trappe. *P*-values indicate statistical significance between values. Percentages gives the change of values for da1 compared to Trappe. Table insert shows a comparison between experiments from 2018 and 2019 in percentage, (Trappe = 100 %), green and red colour designate significant higher and lower values of da1 compared to Trappe. n = 8; bold, significant differences; *, P<0.05; **P<0.01; ***, P<0.001.
